# A small antimicrobial peptide derived from a *Burkholderia* bacterium exhibits a broad‐spectrum and high inhibiting activities against crop diseases

**DOI:** 10.1111/pbi.14506

**Published:** 2024-11-13

**Authors:** Gamarelanbia Mohamed, Ao Ji, Xinyu Cao, Md. Samiul Islam, Mohamed F. Hassan, Yang Zhao, Xing Lan, Wubei Dong, Hongqu Wu, Wenxing Xu

**Affiliations:** ^1^ National Key Laboratory for Germplasm Innovation & Utilization of Horticultural Crops Wuhan China; ^2^ Hubei Hongshan Laboratory Huazhong Agricultural University Wuhan China; ^3^ College of Plant Science and Technology Huazhong Agricultural University Wuhan China; ^4^ Key Lab of Plant Pathology of Hubei Province Wuhan China; ^5^ Hubei Biopesticide Engineering Research Centre Hubei Academy of Agricultural Sciences Wuhan China; ^6^ Department of Agriculture Botany Faculty of Agriculture Al‐Azhar University Cairo 11651 Egypt

**Keywords:** crop disease, plant protection, biocontrol, antimicrobial peptide, antagonistic bacteria, antibiotics

## Abstract

Crop diseases cause significant quality and yield losses to global crop products each year and are heavily controlled by chemicals along with very limited antibiotics composed of small molecules. However, these methods often result in environmental pollution and pest resistance, necessitating the development of new bio‐controlling products to mitigate these hazards. To identify effective antimicrobial peptides (AMPs) considered as potential sources of future antibiotics, AMPs were screened from five bacterial strains showing antagonism against a representative phytopathogenic fungus (*Rhizoctonia Solani*) through the *Bacillus subtilis* expression system, which has been developed for identifying bacterial AMPs by displaying autolysis morphologies. A total of 5000 colonies were screened, and five displaying autolysis morphologies showed antagonism against *R. solani.* A novel AMP with the strongest antagonism efficiency was determined and tentatively named HR2‐7, which is composed of 24 amino acids with an alpha‐helical structure. HR2‐7 has strong and broad‐spectrum antimicrobial activity, tested against 10 g‐positive and ‐negative bacteria and four phytopathogenic fungi by contact culture in plates with minimal lethal concentrations of 4.0 μM. When applied as purified peptide or in fermented *B. subtilis* culture solution, HR2‐7 showed strong controlling efficiency on plants against diverse fungal and bacterial pathogens. Based on current understanding, HR2‐7 is recognized as the first AMP derived from an agricultural antagonistic bacterium. It exhibits wide‐ranging and notable antimicrobial efficacy, offering a supplementary approach for managing plant diseases, in addition to conventional chemical pesticides and antibiotics.

## Introduction

Plant diseases caused by viruses, bacteria and fungi pose significant threats to crops, resulting in substantial losses and compromising the quality and safety of agricultural products (Montesinos, 2007). Among these pathogens, *Rhizoctonia solani* is one of the most prevalent soil‐borne pathogens worldwide, infects a broad host range of crops, and causes significant economic losses in several economically important crops such as maize, rice and soybean (García *et al*., 2006). This pathogen attacks underground plant parts, such as roots, hypocotyls and seeds, while also infecting above‐ground plant parts, such as stems, pods, leaves and fruits (Zhang *et al*., 2012); *Botryosphaeria dothidea* is an important phytopathogenic fungus with a worldwide distribution, which infects numerous plant species, including apple, pear and grape, causing symptoms that include die‐back, stem and shoot blight, gummosis, canker and fruit rot (Yaegashi *et al*., 2012); *Didymella theifolia* is a newly identified fungus that infects tea plants causing tea leaf brown‐black spot disease and affects tea quality leading to a bitter and astringent flavour (He *et al*., 2022); *Botrytis cinerea* is an airborne plant pathogen with a necrotrophic lifestyle, attacking over 200 crop hosts worldwide (Williamson *et al*., 2007); and *Pseudomonas syringae* pv. *tomato* is causative agent of the bacterial speck disease of tomato worldwide and causes severe reduction in fruit yield and quality, particularly during cold and wet springs (Cai *et al*., 2011).

Antibiotics have been proven effective against plant pathogens (Kościuczuk *et al*., 2012; Moravej *et al*., 2018), but their widespread use has led to a decline in control efficacy, fostering the emergence of drug‐resistant pathogenetic strains (Moravej *et al*., 2018; Rathinakumar and Wimley, 2010). As a result of this surge in antibiotic resistance, many infections have become unresponsive to conventional medications (Li and Webster, 2018), prompting a heightened focus on identifying novel antimicrobial genes to counteract bacterial resistance in plant protection (Lindsey *et al*., 2020; Marcos *et al*., 2008). To address the escalating antibiotic resistance in plant pathogens, researchers have turned to exploring new antimicrobials or devising alternative strategies. In recent years, numerous natural and synthetic chemicals have been discovered and employed in plant protection, with antimicrobial peptides (AMPs) emerging as promising candidates against phytopathogens (Amso and Hayouka, 2019). AMPs attract attention for their powerful activity against a wide spectrum of microorganisms, including fungi, bacteria, parasites and viruses (Akaddar *et al*., 2010; Liang *et al*., 2018). This concern has driven scientists to seek novel antimicrobial agents from natural sources, including low‐virulent bacterial strains, exhibiting general antimicrobial activity against clinically significant microorganisms (Ananou *et al*., 2010). AMPs exhibit unique characteristics, including a broad antibacterial spectrum, rapid germ‐destruction capabilities, low bactericidal concentrations, versatility for standalone or combination use and minimal side effects (Hancock *et al*., 2016). These characteristics have motivated scholars to develop methodologies for the isolation and purification of new categories of AMPs and their derivatives across diverse species (Kong *et al*., 2018; Mygind *et al*., 2005). Although recent studies have primarily focused on the extraction, separation, purification and synthesis of AMPs, inquiries have also expanded to include investigations into the mechanisms of resistance (Camó *et al*., 2017). Antimicrobial genes are commonly applied in agriculture, enhancing resistance to plant diseases and contributing significantly to crop variety improvement (Lin *et al*., 2010). Recent publications in esteemed journals have underscored the antimicrobial properties of peptides such as AtR472 derived from *Aegilops tauschii* Cosson, the cationic antimicrobial gene HCAP‐18 identified in Chinese cabbage, and peptide SM‐985 sourced from Teosinte (*Zea mays* ssp. mexicana), demonstrating their efficacy against various plant pathogenic microorganisms (Fu *et al*., 2020; Qutb *et al*., 2020). Moreover, the versatile bacteriostatic effects of AMPs extend their application beyond agriculture, encompassing various fields due to their anti‐inflammatory and other host‐defence features (Souza *et al*., 2020; Zhu *et al*., 2015).

*Bacillus subtilis* has been recognized as a potent instrument within biotechnological research for facilitating the high‐level expression of heterologous proteins (Oumer and Abate, 2017). It is generally considered a non‐pathogenic organism that does not produce endotoxins (Kakeshita *et al*., 2011). The appeal of *B. subtilis* stems from its relatively simple cellular architecture, rapid proliferation, and brief fermentation periods, and it is adeptness at secreting proteins directly into the extracellular environment (Schallmey *et al*., 2004). The products generated by this expression system retain biological activity, necessitating a straightforward downstream treatment (Lee *et al*., 2010; Li *et al*., 2015). Recently, our laboratory developed a genomic library construction method utilizing the *B. subtilis* expression system, asserting its reliability over the *E. coli* expression system (Islam *et al*., 2021). *B. subtilis* not only serves as an effective expression host but also produces beneficial antibacterial compounds, demonstrating potential efficacy against plant diseases (Islam *et al*., 2020). The *B. subtilis* expression system offers additional advantages, including higher yield, absence of product assembly and the ability for continuous cultivation and production (Li *et al*., 2015). In light of these attributes, the current investigation centres on the isolation and characterization of small AMPs using *B. subtilis* expression system.

Considering that most known AMPs were derived from plants, and less from antagonistic bacteria, the study aims to optimize conditions conducive to enhancing the production of AMPs from antagonistic bacteria, ultimately to effectively combat various crop diseases.

## Materials and methods

### Plant samples and bacterial and fungal strains

Rice, maize and rapeseed leaves, as well as roots, were collected from Wuhan, Hubei Province, China, for bacterial isolation. The bacterial strains were initially streaked onto Nutrient agar (NA) medium (10 g tryptone, 3 g beef extract, 5 g NaCl, and 15 g agar per litre; pH 7.0). Fresh single colonies were then inoculated into NA broth. Fungal cultures were cultivated on Potato Dextrose Agar (PDA) medium (200 g dextrose, 17 g agar per litre; pH 7.0) and maintained at 28 °C. The *B. dothide* strain MAO‐1, responsible for pear ring rot disease, was isolated from apple tree branches (*Malus domestica* Borkh. cv. “Fuji”) collected in Shandong province, China (Yaegashi *et al*., 2012). The *D. theifolia* strain CJP4‐1, causing tea leaf brown‐black spot disease, was isolated from tea leaves collected from Zigui county, Hubei province, China (He *et al*., 2022). *B. cinerea* B05.10 known for infecting over 200 crops (Staats *et al*., 2007), *R. solani* AG‐1, and *P. syringae* pv. *tomato* DC3000, a gram‐negative bacterial pathogen isolated from tobacco responsible for the wildfire disease (Sun *et al*., 2021), were obtained from the Key Laboratory of Agricultural Microbiology, Huazhong Agricultural University, Wuhan, Hubei Province, China.

### Bacterial strain identification

The PCR process involved the use of a pair of universal primers: forward primer 27F (5′‐AGAGTTTGATCCTGGCTCAG‐3′) and reverse primer 1492R (5′‐GGTTACCTTGTTACGACTT‐3′), which were procured from T‐Singke in Wuhan, China, and used for amplification of bacterial 16S rDNAs (Reddy *et al*., 2000). The reaction mixture, totalling 50 μL, consisted of 25 μL PCR master mix, 2 μL forward primer, 2 μL reverse primer, 1 μL of DNA template, and 20 μL ddH_2_O. PCR was conducted utilizing a Thermal Cycler (Bio‐RAD, Foster, CA, USA) following thermal program: an initial denaturation at 95 °C for 5 min, followed by 30 cycles of 30 s each at 94, 50 and 72 °C. A final extension of 10 min at 72 °C concluded the process (Islam *et al*., 2020; Zhang *et al*., 2022). The amplified products underwent by electrophoresis on 1.0% agarose gel, and the target bands, approximately 1.5 kb in size, were purified and sequenced by T‐Singke in Wuhan, China. Subsequently, the obtained 16S rDNA sequence was reviewed and compared on the NCBI website (http://www.ncbi.nlm.nih.gov) (Mignard and Flandrois, 2006).

### Antagonistic activity assays

The bacterial solution was inoculated into the plate using a sterilized toothpick at two corners, positioned 3 cm from the centre. Following 24 h, fresh fungal mycelial discs with a diameter of 0.8 cm were placed into the centre of the NA plates. The entire procedure was conducted in triplicate, and plates were then incubated at 28 °C. The extent of fungal growth inhibition was assessed by measuring the length of the inhibition zone (Islam *et al*., 2020). The pathogenic and plant cultures were maintained for the study (Kamaruzzaman *et al*., 2021a, 2021b).

### Construction of genomic libraries

The construction of a genomic library begins with the careful extraction of DNA, followed by the synthesis of genomic DNA as previously described (Doyle and Doyle, 1987). Briefly, this initial step precedes the enzymatic cleavage facilitated by restriction enzymes. DNA is partially digested with *MSe* I and *SauA3* I restriction sites, while the pBE‐S DNA expression vector with *Nde* I and *BamH* I contains all these enzymes from Thermo Scientific company, a critical step enabling subsequent ligation with the vector PBE‐S. The ligated DNA‐vector complex is then introduced into *E. coli* competent cells through a process known as transformation (Das *et al*., 2015). After successful transformation into *E. coli*, the genetic material is further introduced into the *B. subtilis* sck6 expression system, leveraging the unique capabilities of this host for expression studies. These procedural steps, integral to the construction of the genomic library, follow the methodology outlined by Doyle and Doyle, serving as a foundational approach in molecular biology research endeavours (Doyle and Doyle, 1987). A subset of colonies was randomly selected from the genomic library to determine insert sizes with PCR using the general primers associated with vector pBE‐S. The PCR protocol involved an initial denaturation at 95 °C for 5 min, followed by 30 cycles of denaturation at 95 °C for 30 s, annealing at 55 °C for 30 s, extension at 72 °C for 50 s, and a final extension phase at 72 °C for 8 min. To ensure the quality of the genomic library, sequencing analysis alongside gel electrophoresis were employed.

### Screening of genomic libraries

The screening and confirmation processes were carried out as previously described (Kong *et al*., 2018). Briefly, an overnight bacterial culture was plated on Luria‐Bertani (LB) agar plates supplemented with kanamycin at a concentration of 10 mg/L. These plates were then incubated at 37 °C to facilitate the observation of phenotypic outcomes. Following this incubation period, a procedure for cell lysis was implemented (Choi *et al*., 2016). The growth of bacterial colonies was carefully monitored at 12 h intervals over a duration of 48 h, with quantitative assessments of lysed bacterial cells being systematically recorded. To validate the role of specific genes in the process of cell lysis, plasmids extracted from selected bacterial strains were transformed into *B. subtilis* as previously described (Kong *et al*., 2018). Ammonium sulphate precipitation techniques were used to extract a potential protein from the *B. subtilis* expressing system (Zhang *et al*., 2010).

### Antimicrobial activity assay and multivariate data analysis

The antibacterial impact of the antimicrobial peptides was assessed using filter papers as previously described (Xiao *et al*., 2012). Initially, 400 mL of bacterial broth was added to semi‐solid NA (55 °C), and the NA plates were covered before placing the solid NA medium into a 90 mm culture dish. Using tweezers, a piece of 6 mm filter paper was inserted into the top layer of the semi‐solid NA after it had solidified. Subsequently, 20 μL of protein droplets were pipetted onto the filter paper. After the plates had been incubated for 5–8 h at the bacteria's culture temperature, the diameter of the inhibitory zone was determined. Seven types of bacteria were used to test the antimicrobial activity of 29 genes, including the Gram‐positive bacteria *Clavibacter fangii* 1.1999, C. *michiganensis* subsp. *michiganesis* YCK, *B. subtilis* A, *B. subtilis* 905 and *B. subtilis* k1, as well as the Gram‐negative Bacteria *R. solanacearum* R21‐5 and *Xanthomonas oryzae* pv. *oryzae*, XG‐25. Luria‐Bertani (LB) medium was utilized to support the entire bacterial population. Principal component analysis (PCA) of antimicrobial genes was conducted using Minitab software (Pinheiro *et al*., 2021).

### Bioinformatics analysis

#### Peptide characterization

The physicochemical properties, including amino acid composition, hydrophobic ratio, Boman index, and molecular weight, isoelectric point and net charge, were analysed using the APD3 server (Wang, 2015). The secondary structure of HR2‐7 was predicted using the server I‐TASSER (Roy *et al*., 2010). BLAST was utilized to determine the similarity of HR2‐7 to other AMPs in various AMPs databases, including CAMPR3 (Waghu *et al*., 2016), APD3 (Wang, 2015) and dbAMP (Jhong *et al*., 2019). Furthermore, the helical wheel generated by HeliQuest (Gautier *et al*., 2008).

#### 3D structure prediction

The 3D structure of HR2‐7 was predicted by I‐TASSER and the PEP‐FOLD3 (Roy *et al*., 2010), then visualized using UCSF Chimera 1.14rc software. The structure of HR2‐7 was validated by MolProbity (Williams *et al*., 2018), and the helical wheel diagram was designed by HeliQuest (Gautier *et al*., 2008).

#### Synthesize of the HR2‐7 peptide

The GenScript® Corporation (Piscataway, NJ, USA) employed the 9‐fluorenyl methoxycarbonyl (Fmoc) solid‐phase technique to synthesize HR2‐7 peptide. Following synthesis, HR2‐7 peptide underwent refinement to achieve 97.9% purity using high‐performance liquid chromatography (HPLC), and mass spectrometry was utilized to determine its molecular weight. The peptide content was calculated based on its molecular weight and subsequently re‐suspended in ultrapure water, as outlined in the company's peptide solubility test report. It was then stored at −80 °C.

### Minimal inhibition concentration (MIC), minimal bactericidal concentration (MBC) and minimal lethal concentration (MLC) tests

MIC and MBC were determined against 10 bacterial indicators using agar and broth dilution methods (Wiegand *et al*., 2008). Each strain was initially cultured from a single colony in Mueller Hinton Broth (MHB) under two temperature conditions: 28 °C for non‐pathogenic strains and 37 °C for pathogenic strains. The cultures were then diluted in MHB to achieve a target density of approximately 1 × 10^6^ colony‐forming units (CFU)/mL. A primary solution of HR2‐7 peptide was prepared at a concentration of 128 μM in MHB, from which eight serial dilutions were made in a microtitre plate, in serial dilutions of 128, 64, 32, 16, 8, 4, 2 and 1 μM. The bacterial suspension was introduced into each well containing the peptide dilutions, as well as into a growth control well devoid of HR2‐7. Incubation was carried out at respective temperatures of 28 °C for non‐pathogenic bacteria and 37 °C for pathogenic bacteria for a duration of 8 h. MIC was identified as the lowest concentration of HR2‐7 resulting in at least 80% inhibition of bacterial growth compared to the growth control, following the criteria outlined in reference (Wu *et al*., 2014). MBC was ascertained as the lowest concentration of HR2‐7 at which no bacterial growth was observed, as defined in reference (Kang *et al*., 2011).

The MLC was determined by the lowest HR2‐7 concentration that resulted in no bacterial growth in the treated plates. Bacterial suspensions with approximately 1 × 10^6^ colony‐forming units per millilitre (CFU/mL) were produced with 10 μM phosphate buffer for each bacterial indicator (Van de Velde *et al*., 2010). The microtubes were gently inverted every hour during the incubation period. Following incubation, serial dilutions were performed on both treatments and controls. Medium plates in triplicate were used to plate out 100 mL of the appropriate dilution (30–300 CFU per plate). Incubation continued until visible colonies emerged at 28 °C and 37 °C for pathogenic and non‐pathogenic bacterial indicators, respectively. This experiment was independently repeated three times. The microtubes containing the bacterial suspension were exposed to various final concentrations of HR2‐7 peptide (128, 64, 32, 16, 8, 4, 2 and 1 μM). A control microtube was treated with distilled water. Incubate was carried out for 4 h at 28 and 37 °C for pathogenic and non‐pathogenic bacterial indicators, respectively. The microtubes were gently inverted every hour during the incubation period. Following incubation, serial dilutions were performed on both treatments and controls. Medium plates in triplicate were used to plate out 100 mL of the appropriate dilution (30–300 CFU per plate). Incubate continued until visible colonies emerged at 28 and 37 °C for pathogenic and non‐pathogenic bacteria indicators, respectively. This experiment was independently repeated three times.

### Plant disease control tests

Tobacco plants (*N. benthamiana*) were cultivated in a growth room at 28 °C under a 14/10 h light/dark cycle, and their leaves were collected after 5 weeks. Tender leaves of pear (cv. Yuanhuang) and tea (E'cha no.1) were collected in the field, while pear fruits (cv. Yuanhuang) were purchased from a supermarket. The experimental protocol involved treating a bacterial suspension of *B. subtilis* at a concentration of 10^6^ CFU/mL or with synthesized HR2‐7 peptide at a concentration of 4 μM, with distilled water serving as the control. Bacterial suspensions (10 mL) were evenly applied to the leaf or fruit surface to ensure uniform coverage. The bacterial suspension of *P. syringae* pv. *tomato* DC3000 was prepared at a specified concentration of approximately 1 × 10^6^ CFU/mL, following the guidelines outlined in reference (Qutb *et al*., 2020).

Following inoculation, both control and treated plant leaves or fruits were enclosed in nylon bags to create an environment with 100% relative humidity around them. These samples were then placed in a growth chamber set to a regulated photoperiod of 14 h at a temperature of 28 °C until the symptoms appears obviously, as previously described (Qutb *et al*., 2020). At six days post‐inoculation (dpi), the developed symptoms were recorded, and the resulted lesions were measured. This procedure was systematically repeated three times to confirm the consistency and accuracy of the experimental outcomes.

### Stability determination of AMP

The bacterial culture solution of *B. subtilis* sck6 and HR2‐7 at a concentration of 1 × 10^6^ CFU/mL was treated under different temperatures (60, 80, 100 °C) for 10 min, exposed to ultraviolet light at 35 μW/cm^2^ for durations of 10, 20, and 30 min, or adjusted from pH 6 to different pH levels (4, 7, 10). The treated solution was then coated onto pear fruits (variety Huang'guan), and inoculated with mycelial discs of *B. dothidea* under wounded conditions after air drying for 4 h. Sterile double distilled water were involved in parallel instead of the bacterial solution negative controls (CK^−^), and the solution without treatment was involved as untreated controls. The inoculated fruits were incubated at 25 °C with 99% relative humidity for four days. Nine repeats were conducted for each treatment.

## Results

### A *Burkholderia* strain confers strong antagonism to an indicator fungal strain

**Figure 1:**
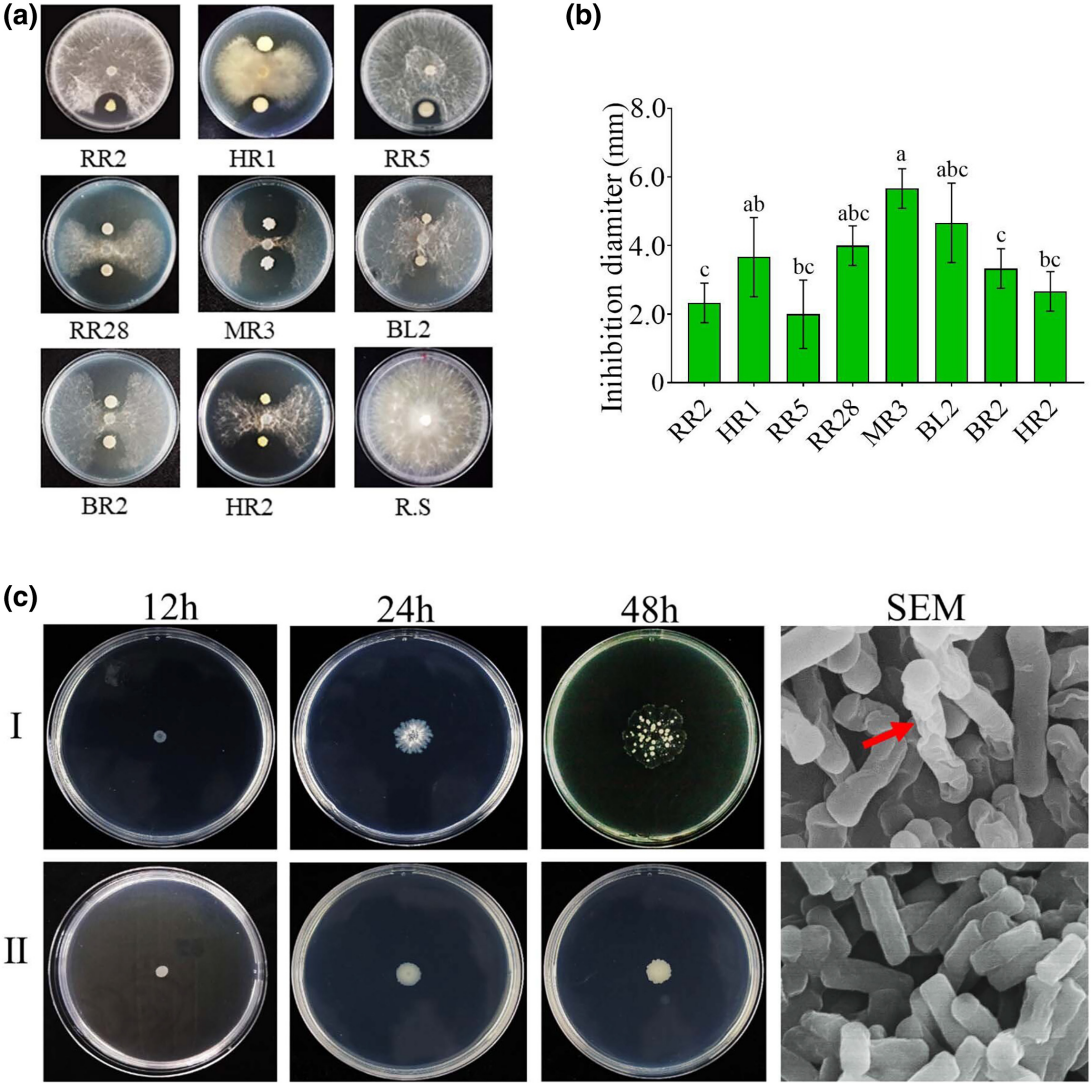
The antagonistic effect of the bacterial strains against *Rhizoctonia solani*. (a) Contact culture of diverse bacterial strains with *R*. *solani* strain AG‐1 in plates. The bacterial strain name is indicated below the plates, and R.S refers to an individual culture of *R. solani*. (b) Bar graph depicting the colony inhibition diameter for *R. solani* strain AG‐1 by dual culture with bacterial strains compared to its individual inoculation. Letters over the error bars indicate a statistically significant difference at the *P* = 0.05 level. (c) The autolytic and normal morphologies of *Bacillus subtilis* cultured on Luria‐Bertani (LB) media and observed under a scanning electron microscope. The morphologies of *B. subtilis* HR2‐7 (I) and sck6 (II) observed at 12, 24, and 48 h after culture on LB media and checked under scanning electron microscope (SEM), respectively.

To isolate agricultural beneficial bacteria, four different plant seedlings (cotton, maize, rice and rapeseed) exhibiting healthy morphology were collected from Hubei province, China. Subsequently, leaves and roots of these seedlings were subjected to bacterial isolation and tested for their antagonistic effects against a phytopathogenic fungus, *R. solani*. A total of 50 bacterial strains were obtained from these samples, among which eight strains (designated as RR2, HR1, RR5, RR28, MR3, BL2, BR2 and HR2) exhibited significant antagonistic effects against *R. solani*. Further identification through BLASTn searches of their 16S rDNA sequences against the NCBI database revealed that the antagonistic strains belonged to the following genera: *Rhizobium* (strain RR2), *Achromobacter* (BL2), *Burkholderia* (HR2), *Agrobacterium* (RR5) and *Bacillus* (MR3, HR1 and RR28) (Figure 1a,b). Notably, *Burkholderia* sp. strain HR2 demonstrated stronger inhibition effects compared to other screened strains, which was selected for screening its potential AMPs. The *B. subtilis* transfectant carrying the HR2‐7 peptide was examined using scanning electron microscopy (SEM). The analysis revealed that the transfectant cells exhibited membrane damage, characterized by the formation of membrane holes, deformation, and lysis, in contrast to those of the control strain without transformation (Figure 1c). This observation suggests that HR2‐7 can disrupt the cellular architecture of the infected bacterial cells.

### Strain HR2 contains novel AMPs

To construct a genomic library, the genomic DNAs extracted from *Burkholderia* sp. strain HR2 along with the vector pBE‐S were digested by the corresponding enzymes, recovered, and purified, respectively. DNA fragments were then inserted into the vector at the enzymatic‐restriction site using T4 ligase and introduced into *E. coli* HST08 cells for multiplication. Subsequently, the plasmids were purified from the *E. coli* cells and transfected into *B. subtilis* sck6 cells after purification, followed by culturing on LB medium plates (Figure S1). The results revealed that approximately 5000 colonies of *B. subtilis* were generated on the plates, with 48 displaying autolysis, which was subsequently assessed by dual culture with *R*. *solani*. Among these, only 25 colonies (or clones) were confirmed to exhibit antimicrobial activity, causing inhibition diameter for the *B. subtilis* colonies ranging from 1.2 to 1.9 cm compared to the control sck6 did (0.9 cm). Inserts in the colonies showing autolysis were sequenced, and their deduced peptides were BLASTp searches with those deposited in the NCBI database, revealing that all these peptides are novel AMPs.

### HR2‐7 exhibited excellent antimicrobial activity as predicted *in silico*

Following the translation of 5000 DNA inserts into amino acid sequences, each sequence was categorized by size: tiny (0–20 amino acids, aa), medium (21–50 aa) and large (over 50 aa). These sequences underwent analysis for (AMP) potential using the CAMPR3 prediction server, which utilizes the Support Vector Machine (SVM) algorithm. From this analysis, 25 sequences were identified with prediction scores ranging from 0.5 to 0.9, indicating potential AMP activity, with HR2‐7 among them. Further validation using additional servers – dbAMP, ClassAMP and iAMPpred – confirmed the antimicrobial potential of HR2‐7 (Table S1), suggesting its efficacy against bacterial targets.

**Figure 2:**
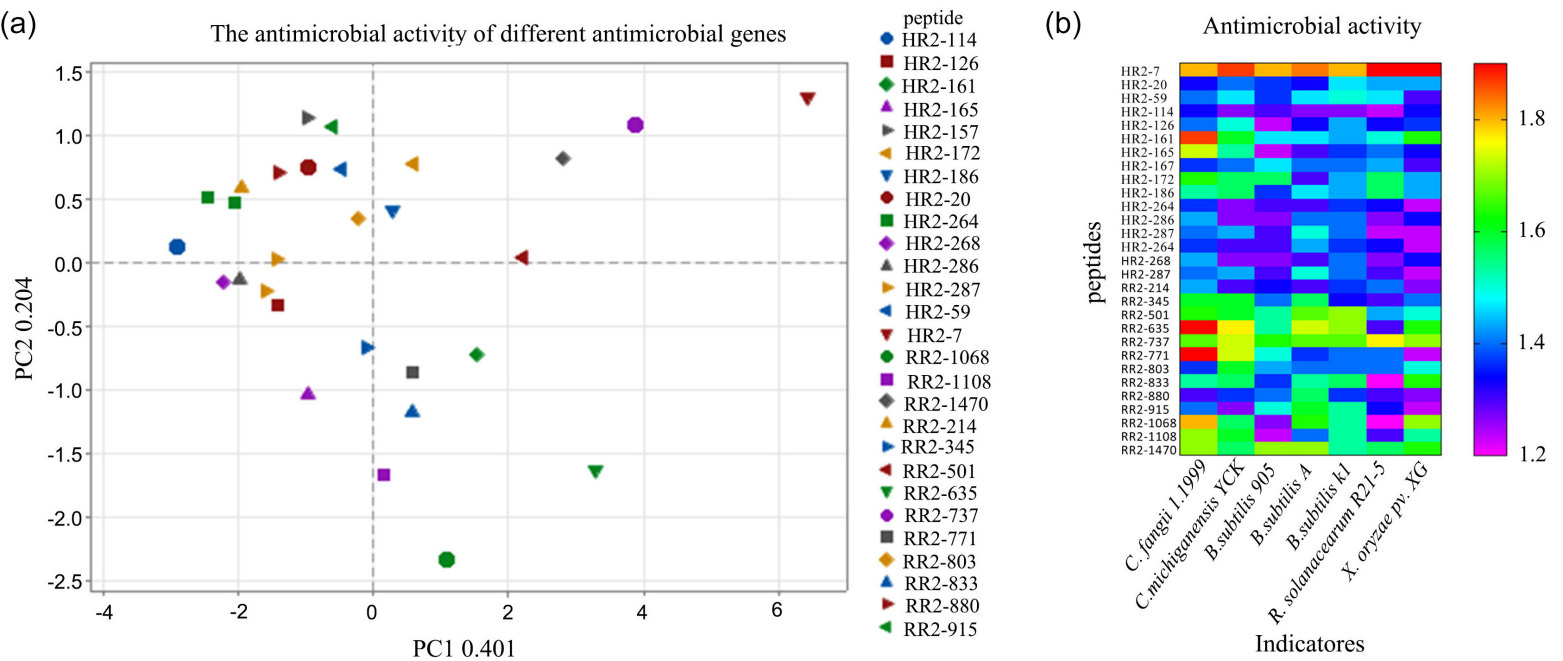
Analysis of antimicrobial activities of diverse antimicrobial genes. (a and b) Principal component analysis (a) and clustered heat map (b) of antimicrobial activity of the identified peptides, respectively.

The extracellular peptides from each potential strain were isolated using the ammonium sulphate precipitation procedure, and their antibacterial properties were assessed. A total of 29 antibacterial peptides, including HR2‐7, were examined, and the results revealed substantial inhibitory effects on both Gram‐positive and Gram‐negative bacterial strains (Figure 2a,b). Specifically, *Clavibacter fangii* 1.1999, *Clavibacter michiganensis* subsp YCK, *B. subtilis* 905, *B. subtilis* A, *B. subtilis* k1, while Gram‐negative bacteria, *Ralostonia solanacearum* R21‐5 and *Xanthomonas* oryzae pv. *oryzae* XG, respectively. Notably, HR2‐7 peptide exhibited significant antibacterial activity compared to other peptides (Figure 2a,b).

### Characterization of HR2‐7

**Figure 3:**
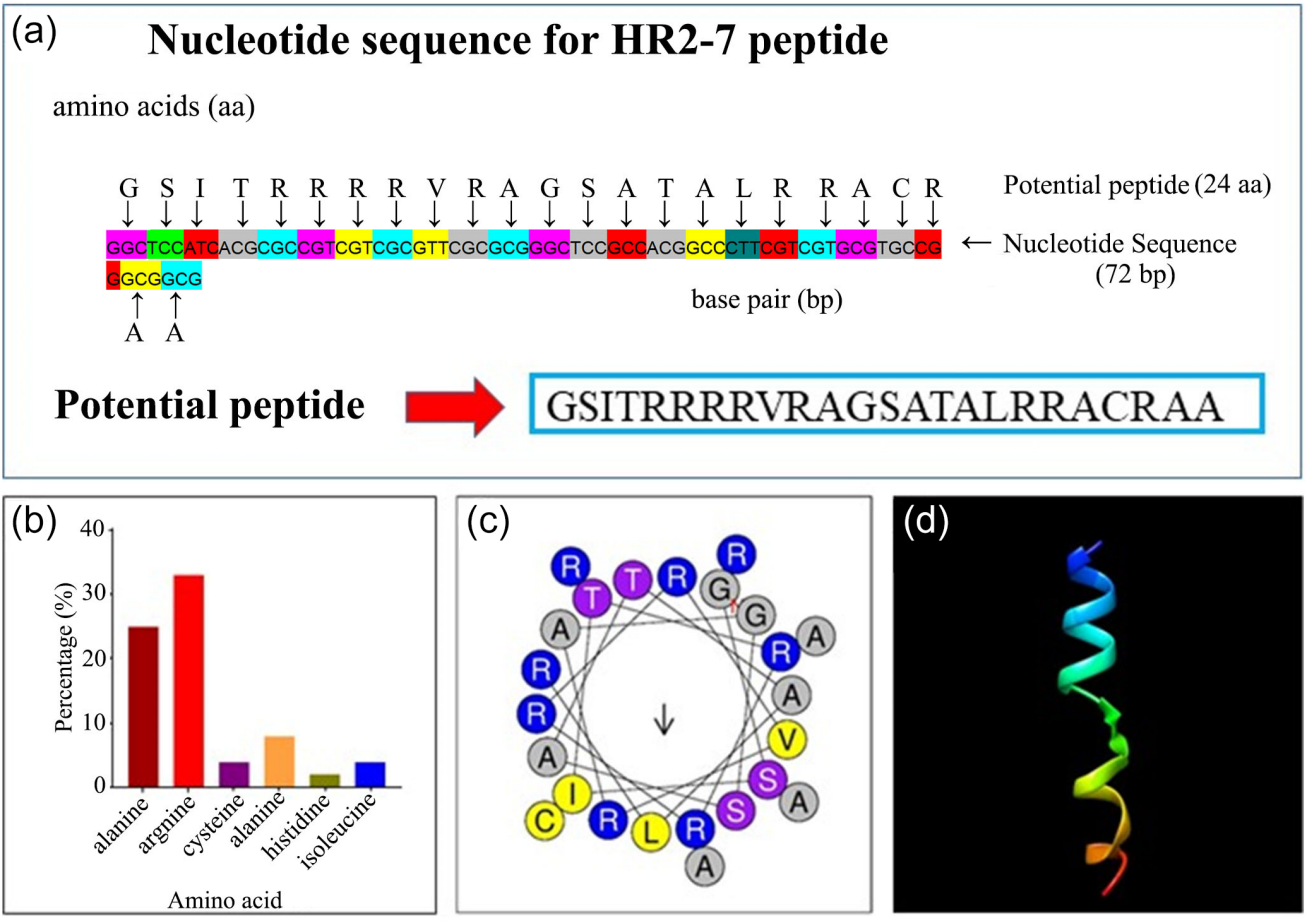
Bioinformatics analysis of HR2‐7 peptides. (a–d) Nucleotide sequence (a), percentage composition of amino acids (b), helical wheel diagram (c), and predicated 3D structure (d) of HR2‐7 peptide, respectively.

According to APD3‐based prediction, HR2‐7 consists of 24 amino acids, including alanine (25%), arginine (33%), cysteine (4%), glycine (8%), histidine (2%) and isoleucine (4%). It exhibits a significantly high hydrophobic ratio (42%) paired with a positive net charge of +8. Moreover, at least six residues are positioned on the same hydrophobic surface, and the Wimley‐White entire residue hydrophobicity parameter is estimated at 4.3 kcal/mol, indicating a strong hydrophobicity. The helical wheel representation of HR2‐7 peptide reveals an amphipathic alpha‐helix (Figure 3c), where the majority of the hydrophobic residues are on the opposite side of the alpha‐helix. The calculated hydrophobic helical moments were 0.233 μH, and the hydrophobicity index was 0.020. The net charge was measured using the Heliquest analysis server (Figure 3b). Furthermore, secondary structure prediction by the I‐TASSER server confirmed the alpha‐helical structure of the HR2‐7 peptide. Integrative assessment involving secondary structure prediction and helical wheel modelling confirmed the amphipathic character of the HR2‐7 peptide by revealing the segregation of hydrophilic and hydrophobic residues on distinct sides of the alpha‐helix. Interestingly, a comprehensive search across multiple databases, including CAMPR3, dbAMP, APD3 and ADAM, reveals no significant identities between HR2‐7 and other AMPs, supporting the notion that HR2‐7 peptide is as a novel AMP.

### Minimum inhibitory concentrations (MICs), minimum bactericidal concentrations (MBCs) and lowest lethal concentration (MLC) of HR2‐7

The antimicrobial efficacy of HR2‐7 was determined by its MICs and MBCs, indicative of the lowest peptide concentration at which no observable bacterial growth occurred against ten phytopathogenic bacteria. These bacteria included Gram‐positive strains, namely *C. fangii* 1.1999, *C. michiganensis* subsp. YCK, *B. subtilis* 905, *B. subtilis* A and *B. subtilis* k1, as well as Gram‐negative strains, *R. solanacearum* R21‐5 and *X*. oryzae pv. *oryzae* XG, along with *B. subtilis and E. coli*. The results unveiled that HR2‐7 peptide exhibited remarkable inhibitory effect, with the lowest effective concentration suppressing 80% of bacterial growth. Specifically, the MIC values ranged from 4 to 16 μM for Gram‐negative bacteria and from 8 to 16 μM for Gram‐positive. Additionally, the MBC values were determined to be 8 to 16 μM for Gram‐negative bacteria and 16 to 32 μM for Gram‐positive bacteria (Table 1).

**Table 1:** Test the minimum inhibitory concentrations (MIC) and minimum bactericidal concentrations (MBC) values (μM) for HR2‐7 peptide against bacterial indicators

Bacterial Indicators	MIC^†^	MBC^†^
*Clavibacter fangii* 1.1999	8	16
*Clavibacter michiganensis* subsp. michiganensis YCKYB1	16	32
*Bacillus subtilis* 168	8	16
*Ralstonia solanacearum* R21‐5	4	8
*Xanthomonas campestris* pv. holcicola 1.1530	8	16
*Xanthomonas oryzae* pv. oryzae XG‐25	4	8
*Pseudomonas syringae* pv. tomato DC3000	4	8
*Erwinia amylovora* E76	16	32
*Escherichia coli* DH5α	4	8

^†^ MIC and MBC tests were performed in accordance with the Broth and Agar method.

The MLC values for all bacterial indicators were established using serial dilutions ranging from 128 to 1 μM of HR2‐7 peptide on plates and cultured with diverse bacterial concentration (1 × 10^6^ CFU/mL). It was determined that an HR2‐7 concentration of ≤4 μM represented the minimal concentration at which complete bacterial death occurred for all bacterial (Figure S2).

### HR2‐7 peptide control diverse diseases

**Figure 4:**
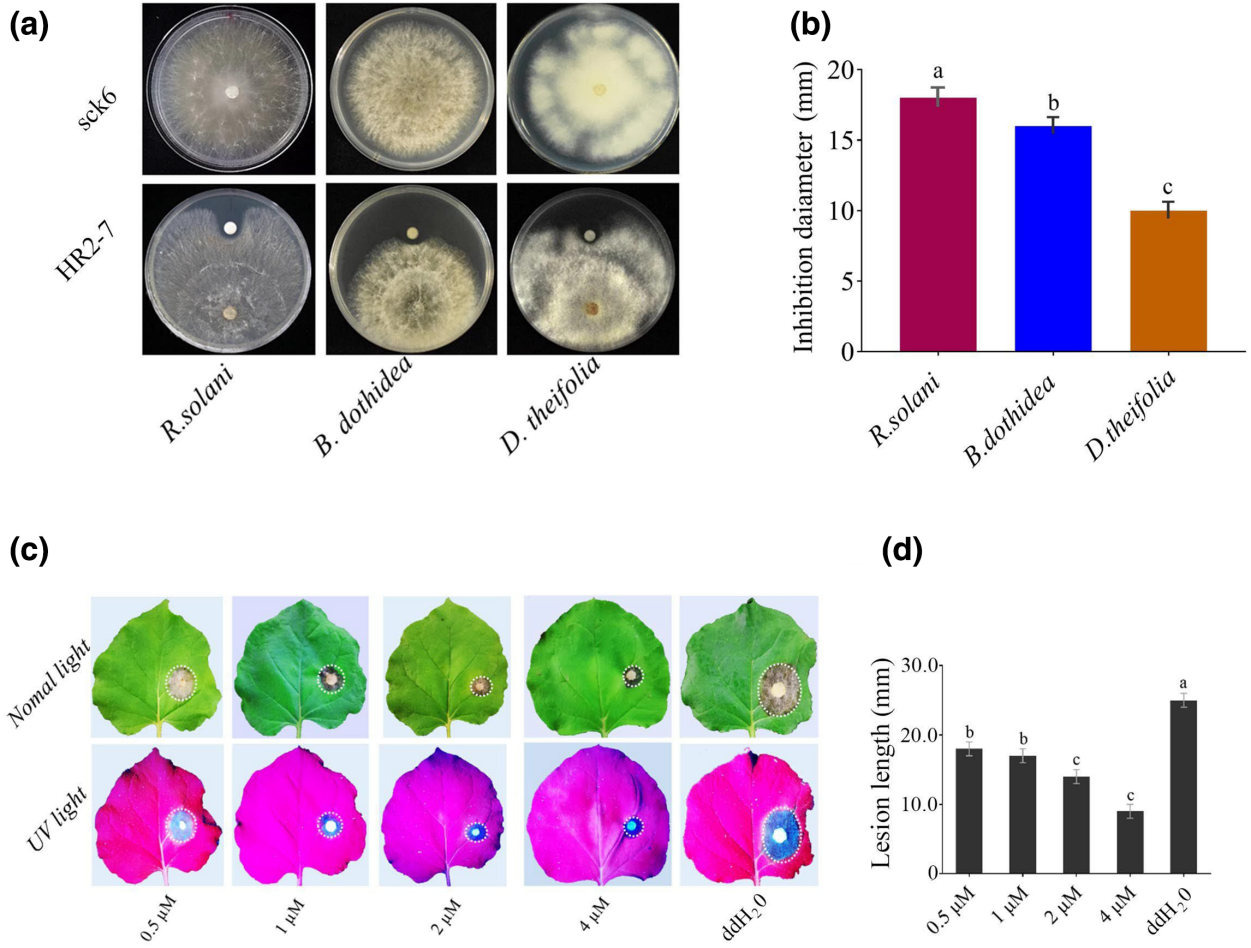
The antagonistic effect of peptide HR2‐7 against fungal strains. (a) Contact culture of HR2‐7 with *R. solani*, *Botryosphaeria dothidea*, and *Didymella theifolia*in on PDA plates. (b) Bar graph for their colony inhibition diameter for fungal strains when dually cultured with HR2‐7 compared with individual inoculation of these fungal strains. (c) The symptoms of tobacco leaves inoculated by *B. cinerea* after treatment with HR2‐7 peptide at serial dilutions (0.5, 1, 2, and 4 μM). (d) Bar graph for the corresponding lesion lengths. The responses were photographed in normal and UV light, respectively. Letters over the error bars indicate a statistically significant difference at the *P* = 0.05 level.

**Figure 5:**
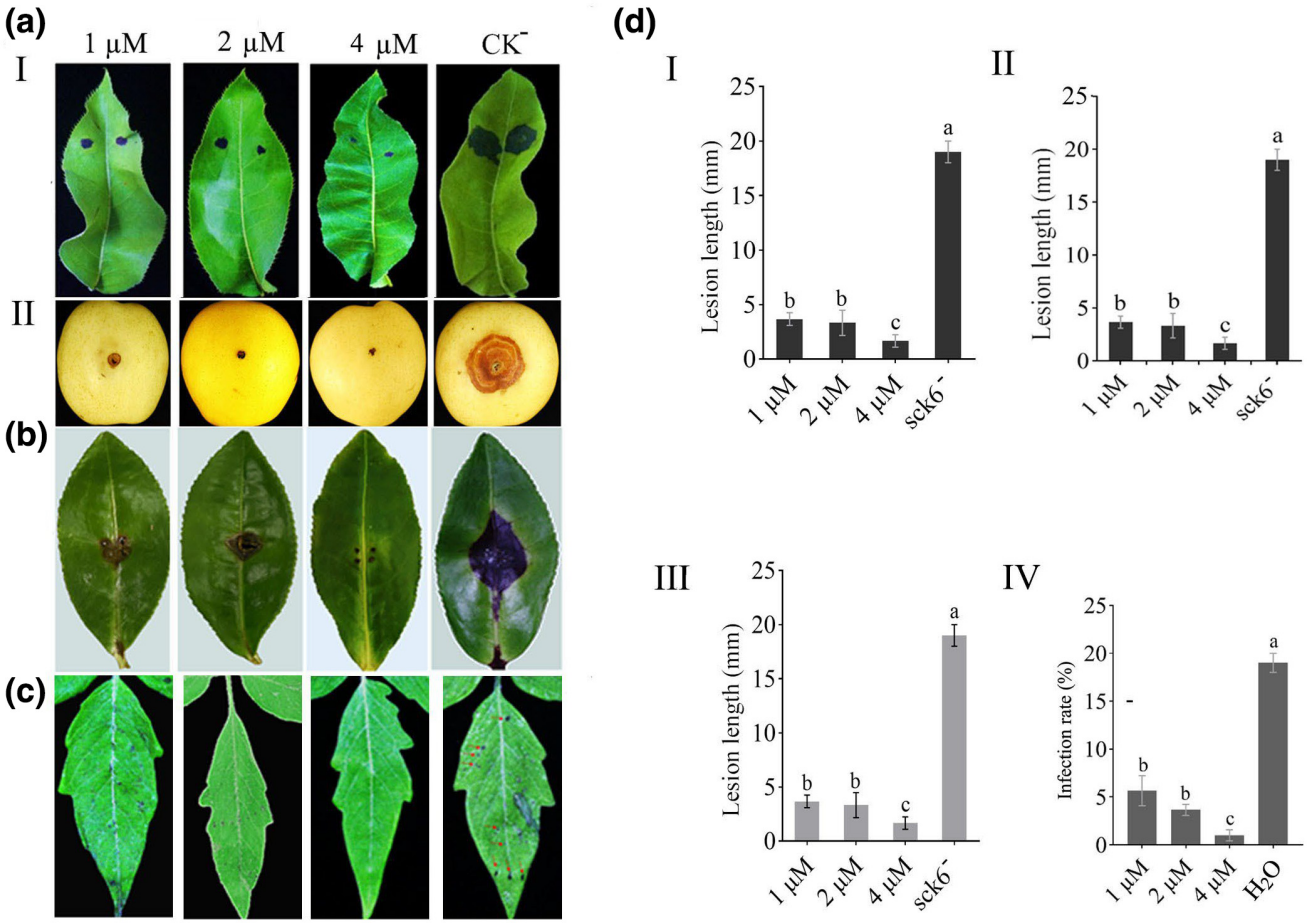
The effect of HR2‐7 against infection of diverse phytopathogens on plants. (a– c) The representative symptoms of pear leave (I) and fruits (II) inoculated by *B. dothidea* strain MAO‐1 (a), of tea leaves inoculated by *D*. *theifolia* strain CJP4‐1 (b), and of tomato leaves inoculated by *P. syringae* pv. *tomato* strain DC 3000 (c), respectively, after treatment with HR2‐7 peptide at serial dilutions (1, 2, and 4 μM). *Bacilius* sck6 (sck6^−^) or sterile water (H_2_O) were involved as negative control (CK^−^). (d) Bar graphs for the corresponding lesion lengths in the pear leaves (I) and fruits (II), tea leaves (III), and tomato leaves (IV). Letters over the error bars indicate a statistically significant difference at the *P* = 0.05 level.

The potential antifungal activity of HR2‐7 peptide was evaluated at a concentration of 4 μM by placing it on filter paper discs, which were placed in contact culture with the mycelial discs of *R. solani*, *B. dothidea* and *D. theifolia*. The results showed that HR2‐7 could inhibit the vegetative growth of all tested fungi (Figure 4a,b). Furthermore, HR2‐7 peptide was applied in plants (including tobacco, tomato, pear and tea) at different concentrations (0.5, 1, 2 and 4 μM) and challenged by inoculating with mycelium discs of corresponding fungal pathogens (*B. cinerea, B. dothidea* and *D. theifolia*) under wounded conditions, respectively. The results indicated that HR2‐7 peptide exhibited significant inhibitory effects against all tested fungi at all tested concentrations. Notably, on *N. benthamiana* leaves inoculated with *B. cinerea*, HR2‐7 peptide reduced lesion size by 10%–30% at 48 h post‐inoculation (hpi) (Figure 4c,d); on detached pear leaves and fruits, HR2‐7 peptide decreased lesion sizes caused by *B. dothidea* by 10%–60% at 5 dpi for the inoculated leaves and by 10%–50% at 4 dpi for the inoculated fruits (Figure 5a,d); on detached tea leaves (*Camellia sinensis* cv. E'cha no.1), HR2‐7 peptide significantly reduced lesions induced by *D. theifolia* by 10%–60% at 7 dpi (Figure 5b,dIII).

Additionally, HR2‐7 peptide was applied to tomato leaves at different concentrations (1, 2, and 4 μM) and challenged by inoculation with a culture solution of *P. syringae* pv. *tomato* DC3000. At 4 dpi, symptoms of leaf spot were observed on the leaves that were sprayed with water, while no symptoms were observed on the leaves treated with HR2‐7 peptide. These results suggest that HR2‐7 peptide is effective in protecting plants against bacterial pathogens (Figure 5c,dIV).

**Figure 6:**
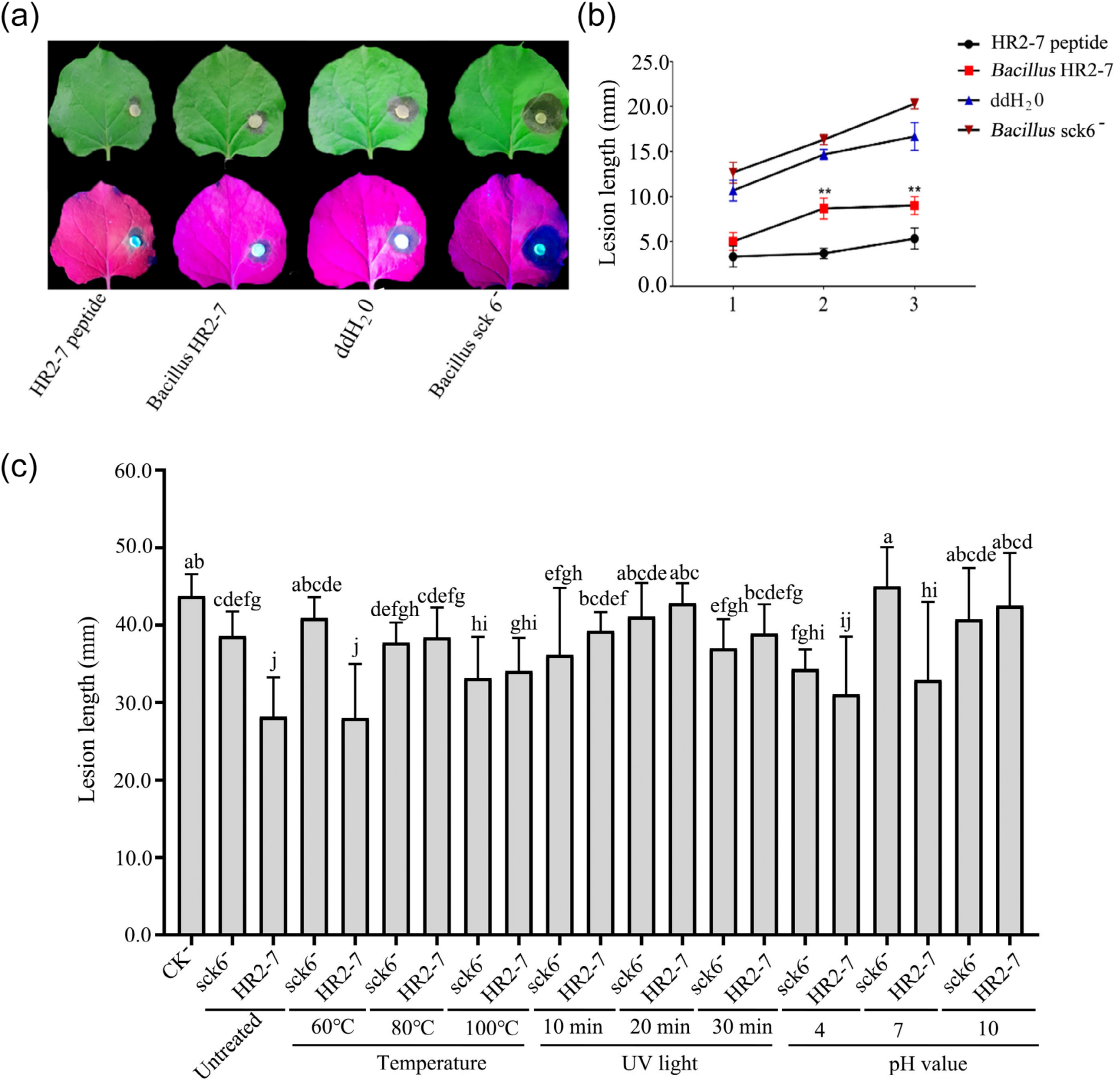
The effect of *Bacillus* HR2‐7 against infection of *B. cinerea* and *B. dothidea* on plants, and its sensitivity to environmental stress. (a, b) The symptoms of tobacco leaves (a) inoculated by *B. cinerea* strain AG‐1 after treatment with HR2‐7 peptide, culture solution, sterile ddH_2_O, and *Bacillus* sck6, and a line graph for the corresponding lesion lengths on the leaves at 1 to 3 dpi (b). Letters over the error bars indicate a statistically significant difference at the **P < 0.01 level. (c) Lesion lengths on pear fruits (variety Huang'guan) resulting from inoculation by *B. dothidea* after treated with sterile water (CK^−^) or culture solution at a concentration of 1 × 10^6^ CFU/mL of *B. subtilis* sck6^−^ (sck6^−^) and *B. subtilis* HR2‐7 (HR2‐7). The solution was treated under different temperatures (60, 80, 100 °C) for 10 min, exposed to ultraviolet light (UV) at 35 μW/cm^2^ for durations of 10, 20, and 30 min, or adjusted from pH6 to different pH levels (4, 7, 10). In parallel, the solution without treatment was involved as controls.

Disease control assays were further conducted using purified HR2‐7 peptide at a concentration of 4 μM and culture solution (1 × 10^6^ CFU/mL) of *B. subtilis* expressing HR2‐7 on detached leaves of *N. benthamiana* under controlled conditions. The results indicated that the culture solution of *B. subtilis* containing HR2‐7 could significantly inhibit the lesions by 10%–30%, which is similar to those treated with purified HR2‐7 peptides compared with those treated with water or *B. subtilis* sck6 devoid of HR2‐7 (Figure 6a,b).

### HR2‐7 is sensitive to some environmental stress

To investigate whether HR2‐7 is sensitive to certain environmental factors, *B. subtilis* HR2‐7 culture solution at a concentration of 1 × 10^6^ CFU/mL was subjected to various treatments. The solution was treated under different temperatures (60, 80, 100 °C) for 10 min, exposed to ultraviolet light (UV) at 35 μW/cm^2^ for durations of 10, 20, and 30 min, or adjusted to different pH levels (4, 7, 10). The treated solution was then evenly applied to pear fruits (variety Huang'guan) and inoculated with mycelial discs of *B. dothidea*. The results indicated that the HR2‐7 culture solution exhibited a strong inhibitory effect on the resulting lesions (2.80–2.82 cm) without thermal treatment or at 60 °C, but nearly lost its inhibition effect on the lesions (3.41–3.84 mm) after treatment at 80 °C or 100 °C, compared to *B. subtilis* sck6 (3.78–4.09 mm). Additionally, as the solution was exposed to ultraviolet light for 10 to 30 min or adjusted to pH 4 or 10, its inhibitory effect was also diminished (Figure 6c). These findings suggest that HR2‐7 has relatively high thermal stability but is sensitive to extreme temperatures, ultraviolet light and acidity or alkalinity.

## Discussion

The significant annual losses attributed to plant diseases and the limitations of pesticide use in many countries have underscored the necessity for novel antimicrobial agents as alternative solutions (Giralt, 2014). While most peptides have been isolated from plants, with only a few originating from bacteria used to disease control (Hassan *et al*., 2021; Qutb *et al*., 2020). This study aimed to isolate novel AMPs from antagonistic bacteria found in the leaves or root of various plants, considering crucial roles in protecting plants against diverse diseases in these organs (Berendsen *et al*., 2012; Chen *et al*., 2020; Finkel *et al*., 2017; Pieterse *et al*., 2016). The peptide HR2‐7 was isolated from a Gram‐negative bacterium, *Burkholderia* sp., which belongs to a group of bacteria known for their diverse metabolic capabilities and their ability to produce a wide array of biochemical compounds, including peptides with unique properties (Depoorter *et al*., 2016). HR2‐7 was identified as a novel AMP since it shares no detectable identities with those isolated from diverse natural sources, including plants, arthropods, microorganisms and animals (Dini *et al*., 2022). Among them, bacterial AMPs have been classified into bacteriocins produced by Gram‐positive and Gram‐negative bacteria (Kumariya *et al*., 2019), with the latter grouped into microcins, colicins, colicin‐like bacteriocins and phage‐tail‐like bacteriocins (Simons *et al*., 2020). Notably, HR2‐7 is distinctly different from known bacterial AMPs, as microcins and colicins are produced by bacteria in the family *Enterobacteriaceae*, Colicin‐like bacteriocins by the *Klebsiella* genus and *P. aeruginosa*, while phage‐tail‐like bacteriocins have structures similar to phage tails (Simons *et al*., 2020), suggesting that HR2‐7 belongs to a new class of AMPs produced by Gram‐negative bacteria.

Numerous computational platforms leverage various attributes and utilize multiple techniques for the prediction and identification of new AMPs (Liu *et al*., 2017). In this study, we utilized various servers to reliably identify potent AMPs, focusing on HR2‐7 predictions. The CAMPR3 server identified HR2‐7 as one of the sequences with the best predictive values, while the ADAM database ranked HR2‐7 as the top AMP. Physicochemical analysis using the APD3 database revealed that HR2‐7 is a 24‐amino acid peptide composed of six different amino acids, with a molecular weight of 2613.03 kDa. Notably, the inclusion of cysteine in HR2‐7 enhances its stability against proteases, indicating increased selectivity for prokaryotes. The compact nature of AMPs, typically consisting of 10 to 100 amino acids with significant membrane activity and amphipathic properties, provides cost advantages in peptide synthesis applications (Reddy *et al*., 2004). However, considering the cost‐effectiveness of simple short sequences in peptide synthesis, HR2‐7's characteristics make it a promising candidate for further development in antimicrobial applications (Ong *et al*., 2014). The positive net charge stands out as a crucial factor influencing the antimicrobial activity of peptides, as exemplified by Bacitracin boasting a net positive charge of (+4). This positive charge plays a pivotal role in the antimicrobial efficacy of these peptides, facilitating interactions with the negatively charged components of bacterial membranes. Cationic AMPs (CAMPs), including the well‐known Bacitracin, typically exhibit a net positive charge within the range of +2 to +8. This characteristic is paramount for their effectiveness in disrupting microbial membranes, ultimately leading to antibacterial effects (Giangaspero *et al*., 2001). HR2‐7 peptide displays an elevated hydrophobicity ratio of 42%, signifying a heightened level of hydrophobic character. Commonly, AMPs feature an excess of positively charged residues on their hydrophilic side, facilitating attachment to the microorganism membrane surface. Simultaneously, the hydrophobic side of AMPs interacts with the bilayer lipid of the cell membrane. This dual interaction contributes to membrane depolarization, inducing cell death through mechanisms such as barrel‐stave pore formation, worm‐hole pore formation or carpet‐type interactions (Sitaram and Nagaraj, 2002). Moreover, the positive Boman index value of HR2‐7, specifically at 4.3 kcal/mol, indicates a favourable protein‐binding potential. This attribute enhances HR2‐7's capability to bind to proteins present in the bacterial cell membrane. In essence, the amphipathic conformation and positive Boman index collectively contribute to the efficacy of ZM‐804 (Hassan *et al*., 2021), illustrating the intricate interplay between charge distribution and hydrophobicity in optimizing interactions with bacterial membranes for potential therapeutic benefits. This hidden interaction of hydrophobic and hydrophilic properties highlights AMPs' different roles in disrupting microbial membranes and elucidates their potential in a wide range of bioactive applications.

The anticipated alpha‐helix secondary structure of the HR2‐7 peptide is closely tied to its expected activity, as observed in previous studies (Park *et al*., 2000). Numerous investigations have identified alpha‐helix structures in various AMPs, emphasizing the significance of this conformation in their functionality (Bonduelle, 2018; Tam *et al*., 2015). The development of an alpha‐helical structure in HR2‐7 serves to enhance its interaction with cell membranes and simultaneously improve membrane permeability to the peptide. Additionally, the physicochemical attributes of HR2‐7 align with those of G17 and G19 peptides documented in earlier research (Gómez‐Sequeda *et al*., 2020). Furthermore, the 3D model of HR2‐7, as suggested by the PROSAII web tool, falls within the favourable structural range, corroborating consistent findings from previous studies (Würz and Güntert, 2017). These collective insights underscore the reliability of the alpha‐helical structure in HR2‐7 and reinforce its potential as an antimicrobial agent with stable and favourable structural characteristics.

According to the MIC and MBC values of HR2‐7 peptide, it demonstrated substantial antimicrobial activity against various bacterial indicators and exhibited diverse effects on both Gram‐positive and Gram‐negative bacteria, as well as phytopathogenic fungi. Significantly, the efficacy of HR2‐7 peptide displayed notable variation between the two bacterial types, with Gram‐negative bacteria proving more susceptible than Gram‐positive bacteria. This observation aligns with findings from ZM‐804 and SM‐985 peptides (Hassan *et al*., 2021; Qutb *et al*., 2020). It is noteworthy that, in most cases, MBC values were greater than MIC values by one to two potencies. In the context of G17 and G19 peptides, their antimicrobial activity against *E. coli* and MRSA was explored through MIC 50, MIC 90 and MBC assessments, despite the lack of similar compositions (Gómez‐Sequeda *et al*., 2020). The varying compositions of cell membranes in each bacterial group may account for the observed differences. The behaviour and capability of AMPs to penetrate layers of peptidoglycan and lipopolysaccharide (LPS) in the membrane, reaching the cytoplasm, could explain the distinctions in MIC and MBC values between the two bacterial groups (Silhavy *et al*., 2010; Yang *et al*., 2006). Following this, the MLC value of 4 μM for HR2‐7 peptide was revealed, indicating that it is lower than both its MIC and MBC values. This disparity in values might be attributed to the testing environment, particularly the composition of the Muller Hinton Broth (MHB) growth media, which is rich in divalent cations. It is known that such components can potentially limit the activity of AMP activity (Farkas *et al*., 2018).

Considering that HR2‐7 exhibits inhibitory activities against a broad range fungal and bacterial strains, it was further applied to control various fungal and bacterial diseases using its pure synthesized peptide and culture solution on detached leaves or fruits, including pear ring rot induced by *B. dothidea*, tea leaf brown‐black spot disease by *D. theifolia*, tobacco grey mould by *B. cinerea*, and tomato bacterial leaf spot by *P. syringae* pv. *tomato*. The results showed that HR2‐7 exhibits the ability to mitigate infections caused by all these fungal and bacterial strains at low concentrations (around the MIC and MBC values), suggesting that it possesses broad‐spectrum and high inhibitory activities against crop diseases. We observed that the peptide significantly curtailed the pathogenicity of *B. cinerea* at different concentrations (0.5, 1, 2 and 4 μM), confirming previous studies on AMPs ZM‐804 and SM‐985 (Hassan *et al*., 2021; Qutb *et al*., 2020). Moreover, HR2‐7 exhibits relatively high thermal stability (up to 60 °C), which will be beneficial for further utilization in the field due to global warming climate. However, HR2‐7 is sensitive to extreme temperatures, ultraviolet light and acidity or alkalinity, differing from most known AMPs, which maintain stable antibacterial activity even after 2 h of UV treatment, exposure to high temperatures up to 100 °C, or in dramatically changed acid–base environments (pH values 3 to 10) (Li *et al*., 2020; Wu *et al*., 2019). We conclude that this discrepancy might be due to the changes in assessment methods, as here we assessed the AMP stability by using the *B. subtilis* solution to inhibit fungal lesions rather than by direct contact culture with the purified peptides and the pathogens on petri plates, since we consider the former method to be closer to the actual application scenario.

Collectively, this study establishes HR2‐7 peptide as a potent antimicrobial agent with a wide range of activity against both pathogenic and non‐pathogenic microorganisms, highlighting its versatility in managing multiple crop diseases across different plant species. AMPs, such as HR2‐7, are gaining prominence in plant protection strategies owing to their low environmental impact and broad‐spectrum activity, offering a sustainable alternative to conventional chemical pesticides. This investigation of HR2‐7 peptide paves the way for its application in safeguarding plants against various bacterial and fungal pathogens, thereby contributing to the development of more resilient agricultural systems.

## Author contributions

W.D., W.X. and H.W. conceived research and designed the experiments; G.M. conducted most of the experiments, A.J. and X.C. conducted the stability assessment, and M.F.H., Y.Z., and X.L. participated in experimental protocols and prepared protocols and regents; M.S.I. and M.F.H improved the English, and W.X. and H.W. improved the English usage and presentation.

## Conflicts of interest

The authors declare no conflict of interest.

## Supporting information


**Figure S1.** (a) DNA extraction for five strains of antagonistic bacteria starting with BR2, RR2, BL2, HR2, and RR5. (b) Gel electrophoresis of pBE‐S plasmid before digestion (c and d) Identification of the pBE‐S plasmid containing inserts inside colonies of *E. coli* (c) and *B. subtilis* (d) after transformation, respectively.
**Figure S2.** The lowest lethal concentration (MLC) tests of HR2‐7 peptide. Ten diverse bacteria cultured on LB media supplemented with 4 μM HR2‐7 peptide and double distilled water (ddH_2_O; control), respectively.
**Table S1.** Informatics analysis of antimicrobial peptide HR2‐7.

## Data Availability

Data sharing is not applicable to this article as no new data were created or analyzed in this study.
