# Supplementary material for: A small antimicrobial peptide derived from a Burkholderia bacterium exhibits a broad‐spectrum and high inhibiting activities against crop diseases
Source: Plant Biotechnol J. 2024 Nov 13;23(2):430–41. doi: 10.1111/pbi.14506 (PMC11772312; doi:10.1111/pbi.14506)
Supplement: Supplementary file 1 — Figure S1. (a) DNA extraction for five strains of antagonistic bacteria starting with BR2, RR2, BL2, HR2, and RR5. (b) Gel electrophoresis of pBE‐S plasmid before digestion (c and d) Identification of the pBE‐S plasmid containing inserts inside colonies of E. coli (c) and B. subtilis (d) after transformation, respectively. Figure S2. The lowest lethal concentration (MLC) tests of HR2‐7 peptide. Ten diverse bacteria cultured on LB media supplemented with 4 μM HR2‐7 peptide and double distilled water (ddH2O; control), respectively. Table S1. Informatics analysis of antimicrobial peptide HR2‐7. [file PBI-23-430-s001.docx]

**Supplementary Figures and Tables**


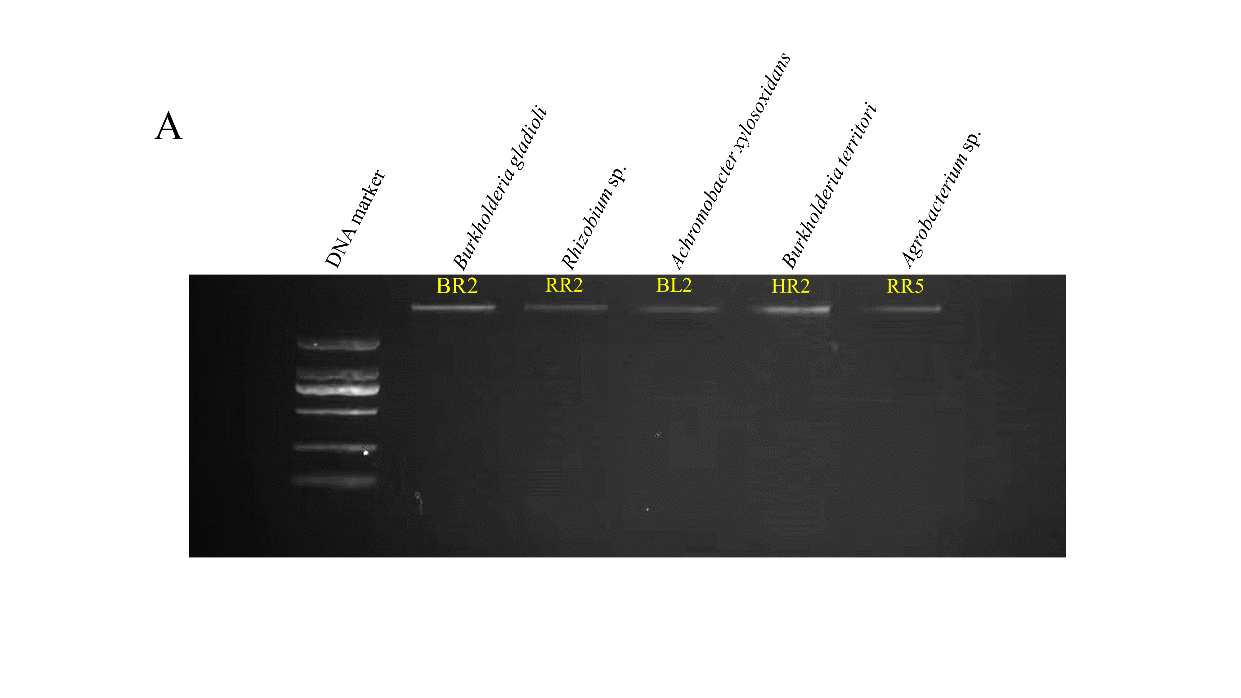

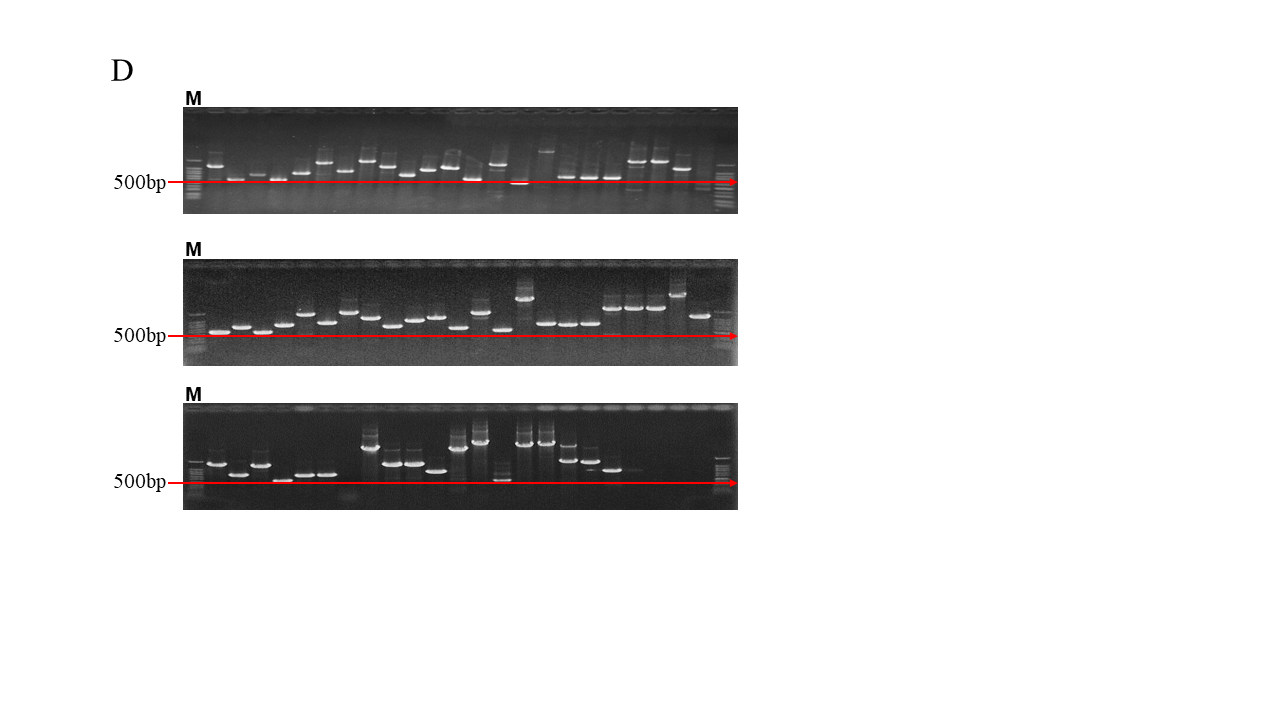

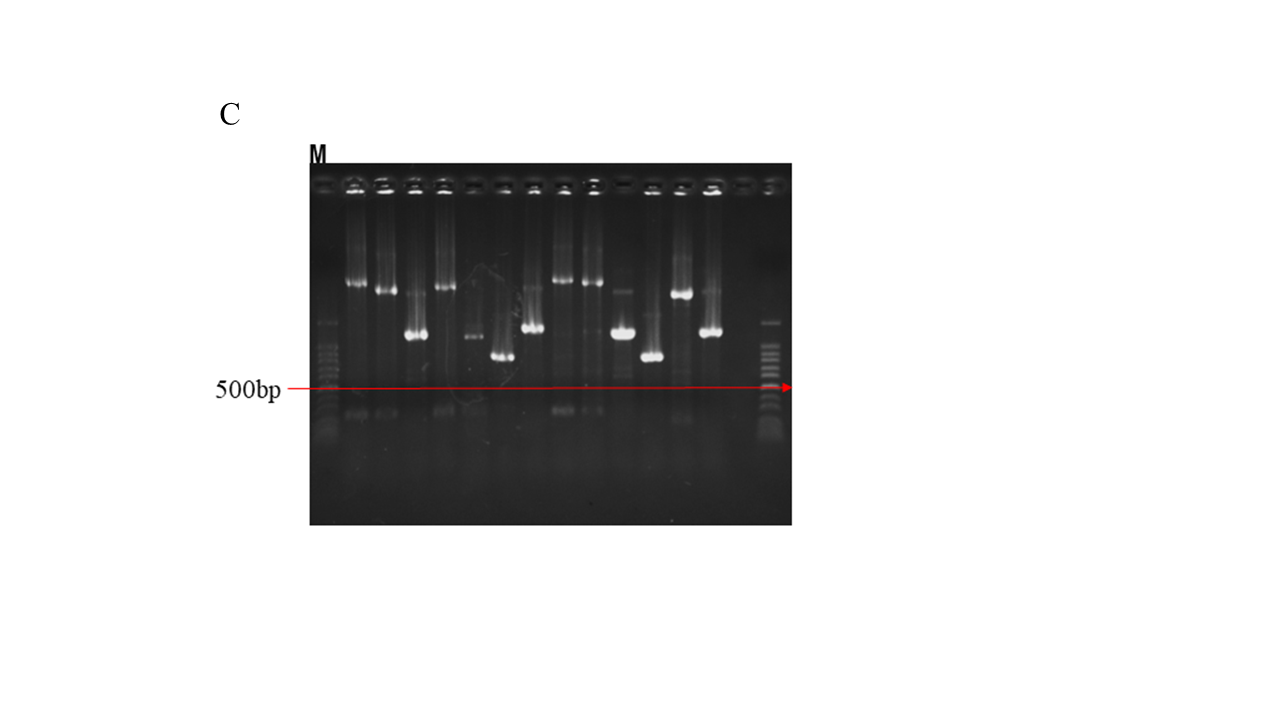
**
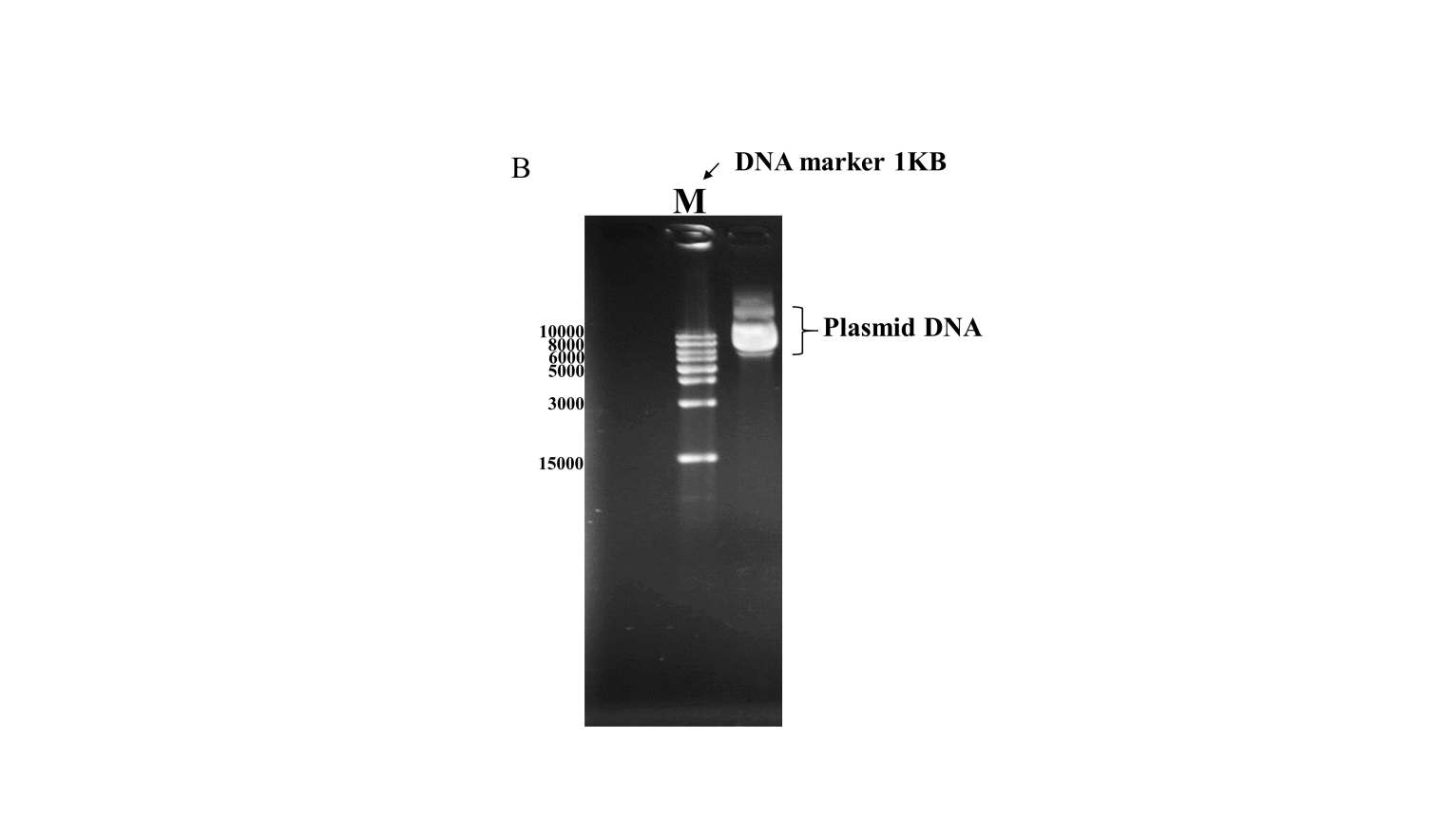
**

**Figure S1** (A) DNA extraction for five strains of antagonistic bacteria starting with BR2, RR2, BL2, HR2, and RR5. (B) Gel electrophoresis of pBE-S plasmid before digestion (C and D) Identification of the pBE-S plasmid containing inserts inside colonies of *E. coli* (C) and *B. subtilis* (D) after transformation, respectively.


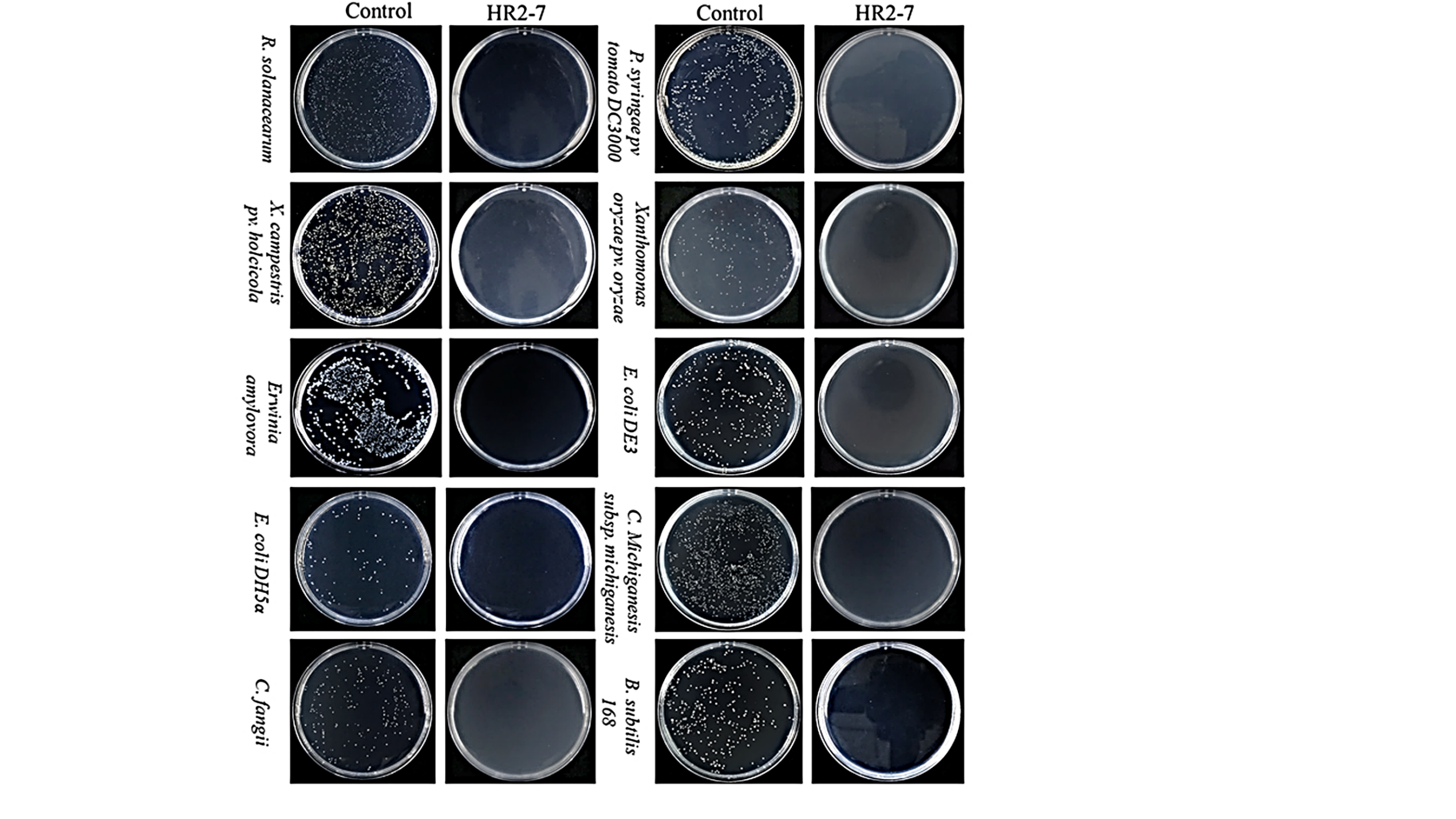


**Figure S2** The lowest lethal concentration (MLC) tests of HR2-7 peptide. Ten diverse bacteria cultured on LB media supplemented with 4 µM HR2-7 peptide and double distilled water (ddH_2_O; control), respectively.

**Table S1** Informatics analysis of antimicrobial peptide HR2-7

| Sever | Algorithms | AMP probability | Class (AMP/NAMP |
| --- | --- | --- | --- |
| DbAMP | RF | 0.86 | Antibacterial |
| ClassAMP | SVM  RF | 0.983  0.904 | Antiviral  Antibacterial |
| iAMPpred | SVM | 0.11  0.087  0.097 | Antibacterial  antiviral  Antifungal |
| AntiBP | SVM  ANN | 1.19  0.98 | Antibacterial  Antibacterial |
